# Supplementary material for: Novel Driver Strength Index highlights important cancer genes in TCGA PanCanAtlas patients
Source: PeerJ. 2022 Aug 11;10:e13860. doi: 10.7717/peerj.13860 (PMC9375969; doi:10.7717/peerj.13860)
Supplement: Supplemental Information 2 [file peerj-10-13860-s002.zip › COHORTS/genes plots/age/2021_8_16_14_9_distribution_cohorts_males_80-84.pdf]

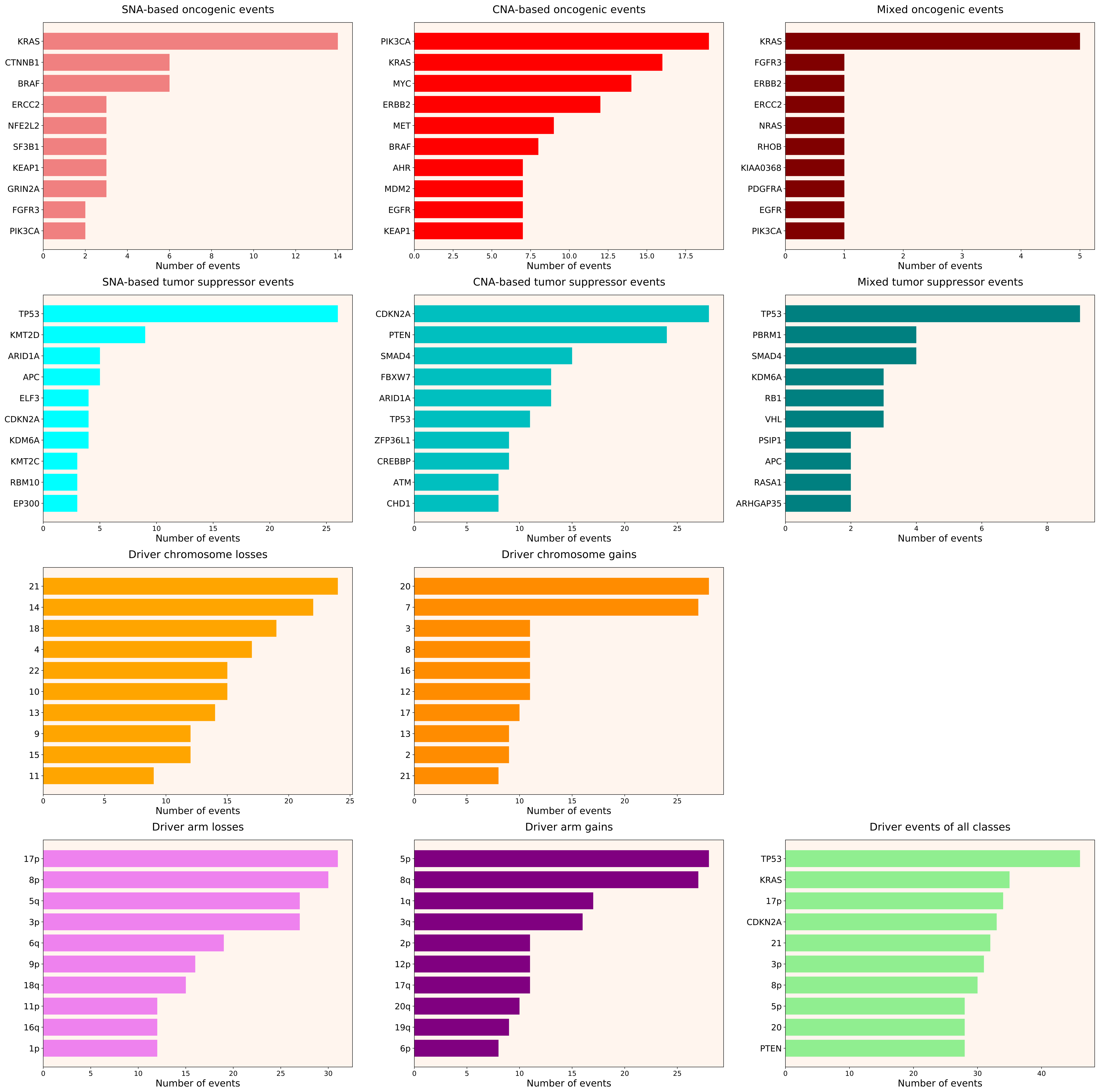
SNA-based oncogenic events
KRAS
CTNNB1
BRAF
ERCC2
NFE2L2
SF3B1
KEAP1
GRIN2A
FGFR3
PIK3CA
0 2 4 6 8 10 12 14
Number of events
CNA-based oncogenic events
PIK3CA
KRAS
MYC
ERBB2
MET
BRAF
AHR
MDM2
EGFR
KEAP1
0.0 2.5 5.0 7.5 10.0 12.5 15.0 17.5
Number of events
Mixed oncogenic events
KRAS
FGFR3
ERBB2
ERCC2
NRAS
RHOB
KIAA0368
PDGFRA
EGFR
PIK3CA
0 1 2 3 4 5
Number of events
SNA-based tumor suppressor events
TP53
KMT2D
ARID1A
APC
ELF3
CDKN2A
KDM6A
KMT2C
RBM10
EP300
0 5 10 15 20 25
Number of events
CNA-based tumor suppressor events
CDKN2A
PTEN
SMAD4
FBXW7
ARID1A
TP53
ZFP36L1
CREBBP
ATM
CHD1
0 5 10 15 20 25
Number of events
Mixed tumor suppressor events
TP53
PBRM1
SMAD4
KDM6A
RB1
VHL
PSIP1
APC
RASA1
ARHGAP35
0 2 4 6 8
Number of events
Driver chromosome losses
21
14
18
4
22
10
13
9
15
11
0 5 10 15 20 25
Number of events
Driver chromosome gains
20
7
3
8
16
12
17
13
2
21
0 5 10 15 20 25
Number of events
Driver arm losses
17p
8p
5q
3p
6q
9p
18q
11p
16q
1p
0 5 10 15 20 25 30
Number of events
Driver arm gains
5p
8q
1q
3q
2p
12p
17q
20q
19q
6p
0 5 10 15 20 25
Number of events
Driver events of all classes
TP53
KRAS
17p
CDKN2A
21
3p
8p
5p
20
PTEN
0 10 20 30 40
Number of events
